# Timeliness of Operating Room Case Planning and Time Utilization: Influence of First and To-Follow Cases

**DOI:** 10.3389/fmed.2017.00049

**Published:** 2017-04-27

**Authors:** Claudius Balzer, David Raackow, Klaus Hahnenkamp, Steffen Flessa, Konrad Meissner

**Affiliations:** ^1^Klinik für Anästhesiologie, Universitätsmedizin Greifswald, Greifswald, Germany; ^2^Kaufmännischer Vorstand, Referat Controlling, Universitätsmedizin Greifswald, Greifswald, Germany; ^3^Lehrstuhl für Allgemeine Betriebswirtschaftslehre und Gesundheitsmanagement, Ernst-Moritz-Arndt-Universität Greifswald, Greifswald, Germany

**Keywords:** operating room scheduling, timeliness, case duration, incision time, resource utilization

## Abstract

Resource and cost constraints in hospitals demand thorough planning of operating room schedules. Ideally, exact start times and durations are known in advance for each case. However, aside from the first case’s start, most factors are hard to predict. While the role of the start of the first case for optimal room utilization has been shown before, data for to-follow cases are lacking. The present study therefore aimed to analyze all elective surgery cases of a university hospital within 1 year in search of visible patterns. A total of 14,014 cases scheduled on 254 regular working days at a university hospital between September 2015 and August 2016 underwent screening. After eliminating 112 emergencies during regular working hours, 13,547 elective daytime cases were analyzed, out of which 4,346 ranked first, 3,723 second, and 5,478 third or higher in the daily schedule. Also, 36% of cases changed start times from the day before to 7:00 a.m., with half of these (52%) resulting in a delay of more than 15 min. After 7:00 a.m., 87% of cases started more than 10 min off schedule, with 26% being early and 74% late. Timeliness was 15 ± 72 min (mean ± SD) for first, 21 ± 84 min for second, and 25 ± 93 min for all to-follow cases, compared to preoperative day planning, and 21 ± 45, 23 ± 61, and 19 ± 74 min compared to 7:00 a.m. status. Start time deviations were also related to procedure duration, with cases of 61–90 min duration being most reliable (deviation 9.8 ± 67 min compared to 7:00 a.m.), regardless of order. In consequence, cases following after 61–90 min long cases had the shortest deviations of incision time from schedule (16 ± 66 min). Taken together, start times for elective surgery cases deviate substantially from schedule, with first and second cases falling into the highest mean deviation category. Second cases had the largest deviations from scheduled times compared to first and all to-follow cases. While planned vs. actual start times differ among specialties, cases of 61–90 min duration had the most reliable start times, with neither shorter nor longer cases seeming to improve timeliness of start times.

## Introduction

Health industries worldwide experience dramatic cost-pressure. Abundant changes in hospital financing require concurrent efforts to increase efficiency throughout entire organizations. Physicians are often at the forefront of such action (1). Operating theaters in particular require thorough planning to streamline management of highly complex and expensive processes. Based on agreed-upon standardized times in Germany, tasks of process analysis and improvement have often been left to anesthesiologists (2, 3).

It has been shown before that timeliness of operating room (OR) planning processes and adherence to scheduled times are crucial for efficient time utilization within large centralized OR units. Determining factors include variation of start times, case durations, and room turnover times. Both turnover times between cases (4, 5) and reducing variation of case duration has been studied in depth, in order to accomplish reliable planning (6). Different models have been applied to individual datasets in order to achieve the basis for reliable and reproducible scheduling of OR cases (7). Ideally, both exact start times and durations of cases are known in advance for each case, surgeon, and supporting team of nurses and anesthesiologists. Ultimately, incentives may be needed to encourage OR timeliness, with key responsibilities shared between all parties involved (8).

Adherence of scheduled first cases to planned times were studied for potential effects on OR utilization (9). However, times for to-follow cases are harder to predict, and data for varying start times and durations of these cases are lacking. The present study therefore aimed to analyze a year of elective surgery cases of a university hospital for deviations from planned times for all electively planned cases in relation to order, duration, and start time. The goal of the present study was to unveil underlying patterns and thereby improve future OR scheduling.

## Materials and Methods

The present observational study aimed to merely describe adherence of case start times to scheduled times in relation to various recorded parameters.

### Data Source

Universitätsmedizin Greifswald is a 900-bed hospital, providing care for three counties and about 250,000 people. It operates a total of 22 ORs and performs about 20,000 surgical procedures per year. All OR scheduling, coordination, and final documentation is carried out *via* a server-based OR management software (myMedis^®^, CARUS GmbH, Norderstedt, Germany). Data are approved on-site and eventually integrated into billing and long-term storage formats. For the purpose of the present study, the database was mined using database software (MS Access, QlikView), with datasets being extracted in readable format (MS Excel) for further processing and analysis.

### Data Processing

All cases saved within 12 consecutive months were extracted and subsequently underwent screening for elective status. All cases that started between 7:30 a.m. and 4:00 p.m. were included. Daytime emergency cases were eliminated from the database in preparation for final analysis. In a next step, cases were assigned to individual specialties and their respective order for the preoperative day planning status. Changes from planned to real start times were noted with respect to change in order. In addition, all respective planning time points (entries into the system) were extracted and assigned to the cases.

### Data Analysis

Cases were categorized for being first, second, or higher in planning status, and according to planned duration, respectively (10). Each case was analyzed for potential deviation from planned start time, and the delay assigned to specific cohorts depending on being early or late, and how much so (in 30-min increments, for practical reasons). In order to exclude irrelevant deviations from scheduled start time, all instances of more than 10-min deviation from scheduled start time were defined as relevant, again in 30-min increments (11). Consequences of these assignments toward the timeliness were analyzed. Finally, the combined influence of the length of the previous procedure and the planning time were analyzed.

## Results

### Case Selection and Processing

A total of 14,014 surgical cases, which were scheduled on 254 regular working days at Universita¨tsmedizin Greifswald between September 2015 and August 2016 were screened for elective status. After 467 emergency cases occurring during daytime had been eliminated from the database, 13,547 elective surgery cases were included in the final analysis.

**Figure 1:**
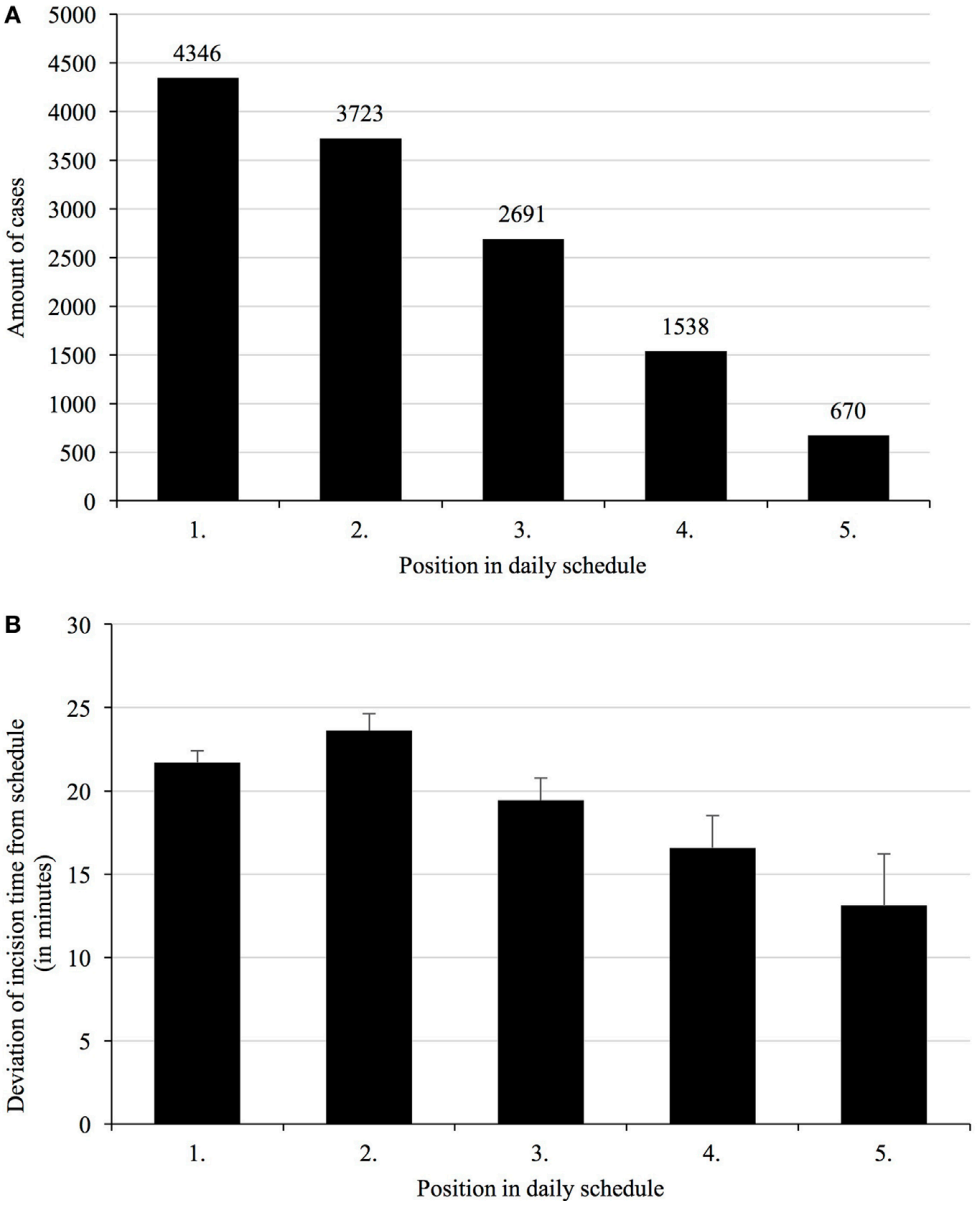
**Total number of cases per position in daily schedule (A), and deviations of incision time depending on the planned order in the daily schedule (B)**. Given the different specialties operating different numbers of cases per day and room, first and second cases represent the majority of cases, with third and to-follow cases taken together still representing a respectable number. Mean deviations of incision times from scheduled times vary with their respective order, with second cases being most unreliable.

On the day before surgery, 4,346 cases were first, 3,723 cases were second, and 5,478 cases ranked third or higher in the daily schedule (Figure 1A). A total of 36% of cases changed their planned start times the day before surgery to the next morning 7:00 a.m., with almost 52% of these resulting in a delay.

### Deviations of Start Times

After 7:00 a.m., a total of 87% of cases started more than 10 min off their originally planned time, which was considered significant for the purpose of this study (Table 1). Out of these, 24% started earlier and 76% later than planned. In relation to the position in the daily schedule, the percentage of cases with a deviation of the incision time of more than 10 min was similar (86%). The percentage of cases starting earlier increased with the position in the schedule (Table 1).

**Table 1:** **Number of cases with a deviation of incision time of more than 10 min depending on the position in the daily schedule**.

Position in daily schedule	Number of cases with deviation	In percentage of all cases	Earlier than scheduled time	Later than scheduled time
1	3,739	86	319 (8%)	3,420 (92%)
2	3,124	84	777 (24%)	2,347 (76%)
3	2,329	86	779 (33%)	1,550 (67%)
4	1,319	86	499 (37%)	820 (63%)
5	582	86	238 (40%)	344 (60%)
All cases	11,836	87	3,066 (25%)	8,770 (75%)

*The relative number of cases with relevant deviations of incision times from schedule remains constant over different positions in the daily schedule. The distinction between earlier and later cases is a relative increase in cases started earlier than scheduled*.

Deviations of start times for first cases was 15 ± 72 min, for second cases 21 ± 84 min, and for all to-follow cases 25 ± 93 min, when compared to preoperative day planning. These times were different when compared to same-day planning status with 21 ± 45 min for first cases, 23 ± 61 min for second cases, and 19 ± 74 min for all to-follow cases (Figure 1B). The morning status includes all overnight changes to the schedule and was henceforth used as reference.

### Deviations of Case Durations

**Figure 2:**
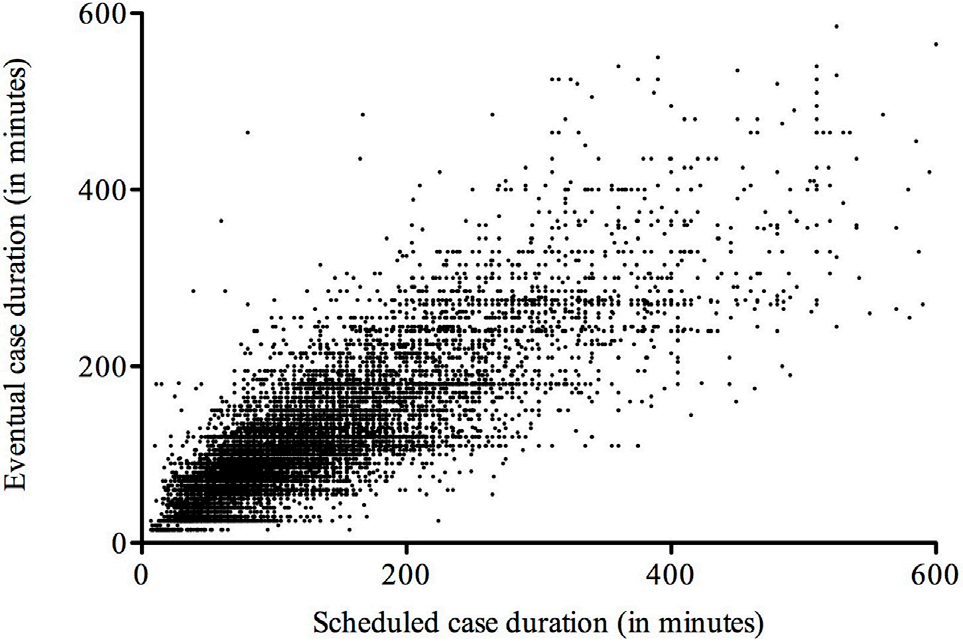
**Deviation of case durations from schedule**. Scheduled case durations deviate from data documented in the operating room system, with short- and medium-length cases obviously being somewhat more reliable than longer ones.

**Figure 3:**
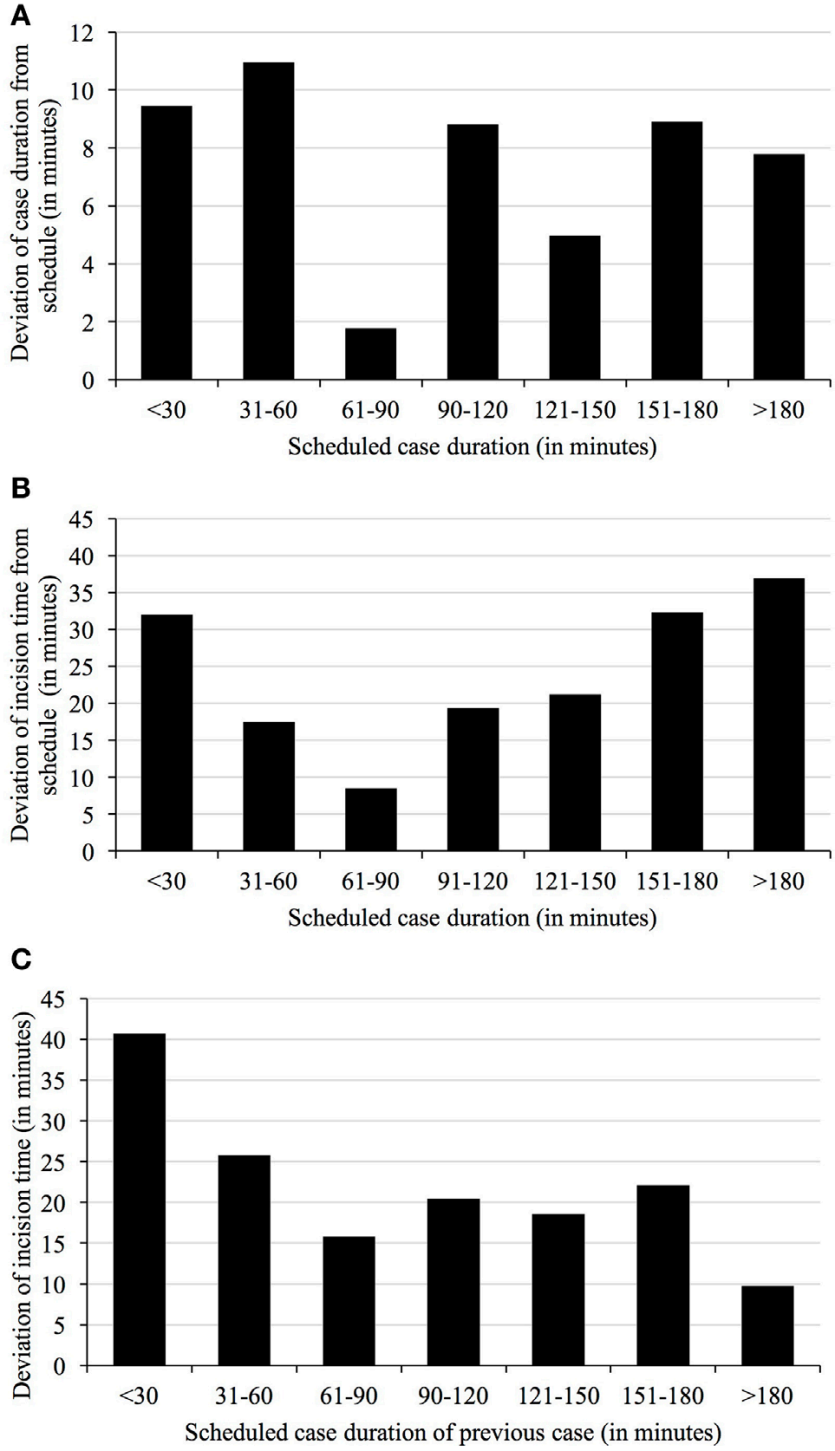
**Deviation of case duration (A) and incision times from schedule depending on duration of scheduled cases (B) and duration of previous cases (C)**. The duration of cases just over an hour in length are most correctly predicted by surgeons **(A)**. Mean deviations of start time from schedule vary with duration of the scheduled cases, with medium-length cases being the more reliable than either short or extra-long cases **(B)**. Mean deviations of start time from schedule also vary with duration of the previous cases, again with medium-length cases being the more reliable than either short or extra-long cases **(C)**.

The duration of surgical cases deviated from the scheduled time allotted to do the case (Figure 2), with shorter procedures having fewer deviations on average. Delays in start times in relation to schedule obviously build on each predecessor (Figure 3A). First cases, therefore, influence all to-follow cases, assuming the duration of cases is planned properly. Thus, duration of cases is equally important for OR time utilization and usually translates directly into the to-follow start times.

Deviations in start time were also related to duration of the procedure (Figure 3B). Cases of 61–90 min in length were found to be most reliable with respect to starting at planned time (mean deviation 9.8 ± 67 min compared to the 7:00 a.m. plan), regardless of order.

### Amalgamation of Start Times and Durations

The timeliness of cases in the daily schedule also depends on the compliance of ORs with scheduled case durations. In response to the prolongation of previous cases, incision times of following cases may be delayed also. Cases following after short (less than 30 min planned duration) cases display the highest deviations (means of more than 40 min, Figure 3C). In contrast, incision times after cases with estimated durations between 61 and 90 min and more than 180 min were more accurate (16 ± 66 and 10 ± 82 min).

### Influence of Planning Time on Start Time Accuracy

Assuming that every elective surgery should be scheduled not later than 4:00 p.m. on the previous day, the influence of the day of planning in relation to the timeliness was analyzed. The average case was planned 20 days in advance, and the deviation of procedure start times increased with the time difference between planning and operation days (mean 21.5 min, shortest deviation 13.5 min for 2 days difference, longest deviation 27.6 min for more than 49 days difference). In relation to the planned length, the 61–90 min cases showed the shortest duration of planning in advance and also the lowest deviation of start times.

## Discussion

The present study analyzes a cohort of elective surgery planning data of a single center over 1 year with respect to potential influence on timeliness of case start times, number of cases done in a room per day, and inherently, time utilization. In addition to known factors influencing such outcome, like start time of the first case, the study addressed factors like buildup of delay, duration of cases, and the co-play of delayed incision and planning errors with respect to duration of earlier cases. The study intends to empower OR managers to base their decisions on more evidence than there is currently available (2).

The number of scheduled cases starting more than 10 min later than originally scheduled is overwhelmingly high. This is particularly important for first cases, which have been shown before (9, 12). Start times for cases starting second or later show sum effects of both early and late start times. However, this does not result in increased timeliness. Cases starting second tend to have the longest delays, compared to first and all to-follow cases. Whether this phenomenon is in itself predictive of poor planning quality warrants further investigation. Nevertheless, all cases following the second one build on the duration of previous cases as well as turnover times, with potentially additive effects for start times. Interestingly, both start times and durations of later cases tend to be no less accurate estimates than for earlier cases, since the mean deviations from scheduled start times decreased from the third case onward, which has not been shown before.

Planning a case in advance does not “protect” it from starting late—compared to recently scheduled cases. Even though there is no evidence in the literature supporting this finding, both OR managers and surgeons seem to adhere to long-term planning. Deviations from start time are also associated with deviations in duration of a case, with both parameters being lowest in the group of 61–90 min. Estimates of surgeons regarding prospective case durations are known uncertainties, which are not readily accessible to predictive modeling (13).

Taken together, start times for elective surgery cases are planned overly optimistic for the majority of cases, with first and second cases being in the highest mean deviation category. Second cases are not more predictive of planning success than to-follow cases in the institution analyzed. While planned vs. actual start times differ widely over surgical specialties, neither ultra-short nor extra-long cases seem to ease timeliness of case start times. Early planning of medium-length cases results in the most predictive procedure in terms of start time and duration of the case.

## Ethics Statement

The data analysis was performed as a measure of quality control. No individual patients or providers were analyzed, and there was no assignment to different groups or change in standard of care.

## Author Contributions

CB and KM designed the study, analyzed the data, wrote the manuscript, and approved the final manuscript version to be published. DR helped with study design, data acquisition and analysis, and manuscript preparation and approved the final manuscript version to be published. KH helped with study design and data acquisition and analysis, critically revised the manuscript, and approved the final manuscript version to be published. SF helped with study design and data analysis, critically revised the manuscript, and approved the final manuscript version to be published. All authors agreed to be accountable for all aspects of the work in ensuring that questions related to the accuracy or integrity of any part of the work are appropriately investigated and resolved.

## Conflict of Interest Statement

The authors declare that the research was conducted in the absence of any commercial or financial relationships that could be construed as a potential conflict of interest.
